# Describing pre‐appointment written materials as an intervention in the context of children’s NHS therapy services: A national survey

**DOI:** 10.1111/hex.13180

**Published:** 2020-12-10

**Authors:** Samantha Armitage, Elaine McColl, Niina Kolehmainen, Tim Rapley

**Affiliations:** ^1^ Sheffield Children’s NHS Foundation Trust Sheffield UK; ^2^ Newcastle University Newcastle upon Tyne UK; ^3^ Northumbria University Newcastle upon Tyne UK

**Keywords:** behaviour change, children's therapy, health service delivery, written materials

## Abstract

**Context:**

Pre‐appointment written materials, including letters and leaflets, are commonly used by healthcare organisations to deliver professional‐patient interactions. The written materials potentially change patients’ knowledge and behaviour as part of a healthcare intervention but have received little investigation.

**Objective:**

To describe the content of pre‐appointment written materials through a behaviour change intervention perspective.

**Design:**

Mixed methods study with an online questionnaire about pre‐appointment written materials and an analysis of actual materials. Questionnaire data were analysed descriptively and pre‐appointment materials by qualitative framework analysis.

**Setting and participants:**

Children's community/outpatient occupational therapy, physiotherapy and/or speech and language therapy services across the UK. Service managers/clinical leads provided data.

**Intervention:**

Pre‐appointment written materials.

**Results:**

Questionnaire responses were received from *n* = 110 managers/clinical leads from *n* = 58 NHS organisations. Written materials (*n* = 64) were received from *n* = 24 organisations. Current materials are used by therapy services as a conduit to convey the therapy service's expectations related to: accessing the service, decision‐making about care and help‐giving. The materials enrol the parent and child to the therapy services’ expectations by behaviour change techniques. The materials configure the parent/child expectations, knowledge and behaviour towards the therapy services’ operational procedures.

**Conclusion:**

Pre‐appointment written materials configure patients to organisations’ operational procedures. The written materials currently lack support for parent/child empowerment, shared decision‐making and self‐management to improve health.

**Patient Contribution:**

Four parents of children accessing therapy services were involved in the study. The parents shared their experiences to highlight the importance of the topic and contributed to the final research design and methods.

## 1 INTRODUCTION

National Health Service (NHS) Trusts in the UK each spend an estimated £12 000‐£87 000 per month to post between 48 000 and 800 000 letters to patients.^1^ Many of these letters will be delivered pre‐appointment to inform patients about the appointment and the service being offered. Pre‐appointment written materials are a form of healthcare interaction, an intervention that takes place before the initial face‐to‐face appointment.^2^ Pre‐appointment written materials represent a significant investment in written interactions on the part of NHS organisations, but their potential role in supporting patient health is poorly understood.^2^ Little is known about current pre‐appointment written materials and how they contribute to quality of care and patient health.

In public health, pre‐appointment letters inviting patients to screening programmes have been developed by drawing on behavioural theory, principles and techniques to enhance letter content and formatting. In the UK, Sallis et al^3^ enhanced invitation letters to the NHS Health Check by: simplification (shortened content, improved readability and reduced complexity); behavioural specificity using behavioural instruction and concrete statements; personal salience, using formatting techniques and lexical choices; and behaviour change techniques of action planning and prompts.^3^ Similarly, ten Hoor et al^4^ enhanced invitation letters to chlamydia testing in the Netherlands by increased personal relevance of informational content to bolster patients’ motivation, simplified content to increase patients’ ability to process the information, and information tailored to patients’ risk perception, attitude, moral norms, social influence and response efficacy and self‐efficacy^4^ found in previous research. Both studies tested the interventions in randomised controlled trials but with opposing results. Sallis et al^3^ found the enhanced letters were significantly associated with increased patient attendance at NHS Health Checks but ten Hoor et al^4^ found no differences in uptake of chlamydia testing between those receiving the enhanced and standard letters. Differences in the health context between the two studies, that is cardiovascular health^3^ and sexual health^4^ and target behaviours, that is attending an appointment^3^ and requesting, using and returning a test kit^4^ may account for differences in findings but it remains unclear if theory‐informed pre‐appointment written materials can positively impact on health behaviours. The above studies specifically focus on patient letters despite the letters being only one part of a package of materials sent to participants. Leaflets^3^ and websites^4^ were also included in the package of materials but were not enhanced. Developing the whole package of written materials into an active health intervention may increase the likelihood of positive behaviour change and health outcomes.

Our study explores current pre‐appointment written materials in children's therapy services in the UK, including, but not limited to, letters. Following the Medical Research Council's guidance on intervention development,^5^ our study aims to characterise the materials as an intervention, including delineation of key intervention components and mechanisms, and to describe the context of children's therapy services in the UK in which these interventions are situated.^6^ A detailed description of current materials is an important step towards developing pre‐appointment written materials into a targeted health intervention to deliver high‐quality care and target health outcomes.

## 2 METHODS

A mixed methods study of children's NHS therapy services in the UK was conducted. The study was approved by the Health Research Authority (HRA) (ref: 245988, 01/10/2018) and was exempt from NHS Research Ethics Committee review because the research involved solely NHS staff and not patients.

The sampling frame for the survey was a database of 144 NHS organisations across the UK and their respective children's occupational therapy, physiotherapy and speech and language therapy services.^7^ The database was developed from Internet searches, expressions of interest from the National Institute for Health Research (NIHR) Clinical Research Network (CRN) and personal networks of the research team. The Royal College of Occupational Therapists: Children, Young People and Families Specialist Section, The Association of Paediatric Chartered Physiotherapists, and the British Academy of Childhood Disability also disseminated the survey to their members.

Inclusion criteria for participants were as follows: managers or leaders of (a) NHS provider/commissioned community and/or outpatient services that (b) included occupational therapy, physiotherapy and/or speech and language therapy (c) for children in the UK. Multi‐professional services (eg wheelchair services, child and adolescent mental health services) were included if they included an occupational therapist, physiotherapist, or speech and language therapist. Exclusion criteria were as follows: adult‐only (16 years +) services; services without at least one of the eligible therapy professions; and inpatient therapy services. The HRA approved participant information sheet was provided to participants online prior to the questionnaire and consent was inferred from questionnaire completion.

Data on the use of pre‐appointment written materials in children's therapy services were collected through an online questionnaire based on the domains of the Template for Intervention Description and Replication (TIDieR).^8^ Initial question items and response options were developed by two authors (SA and NK) from clinical experience and a review of published evidence related to pre‐appointment interventions.^9^ The questionnaire was further refined by a process of peer review and piloting until a final set of items and response options related to ten TIDieR domains were agreed^7^ (see Appendix S1). The final questionnaire was administered online using Qualtrics^©^,^10^ with a link to the questionnaire provided in an email invitation to participate. Data were collected from October 2018 to February 2019.

The online questionnaire included a section on the characteristics of the responding therapy services, comprising: the number of therapists in the team, whole time equivalent staff, number of children known to the service, number of referrals per month, number of weeks wait for an initial appointment and non‐attendance rates.

Data on current pre‐appointment written materials were collected by requesting samples of actual, currently in use materials from the participating services. Participants submitted the samples by email. Written comments or additional information related to the materials that participants submitted in their email correspondence with the documents was retained and included in analysis.

The questionnaire data were transferred from Qualtrics©^10^ to Statistical Package for Social Sciences (SPSS, version 25)©.^11^ Data cleaning and data entry were completed following a data dictionary and analysis plan.^7^ Univariate descriptive analysis was completed. Questionnaire data were primarily categorical (Table 1a) resulting in modal values and percentages to represent the data. For all continuous variables, the null hypothesis that the data followed the Normal Distribution was rejected based on the Shapiro‐Wilk test and visual inspection of the distribution of values. The median, interquartile range (IQR) and range were therefore used to represent continuous variable data (Table 1b).

**TABLE 1:** Characteristics of therapy services

(a)				
Variable (*n* responses)	Questionnaire respondents (*n* = 110)		Sample of written materials (*n* = 33)	
	*n*	% of cases	*n*	% of cases
UK region (*n* = 110): Scotland	5	4.5	1	3
England	97	88.2	29	88
Wales	6	5.5	3	9
Northern Ireland	2	1.8	0	0
Services (*n* = 105): Universal	14	13.3		
Hospital based	36	34.3		
Community based	89	84.8		
Specialist centre	13	12.4		
Professions (*n* = 98): Physiotherapy	57	58.2	10	30
Occupational therapy	48	49.0	11	33
Speech and language therapy	36	36.7	8	24
Other	18	18.4	4	12
Respondents Role (*n* = 100)				
Service manager	31	31.0		
Clinical lead	55	55.0		
Therapist	35	35.0		
Other	9	9.0		
Size of population served (*n* = 96)				
<150 000	10	10.4		
151 000‐250 000	12	12.5		
251 000‐350 000	18	18.8		
351 000‐450 000	8	8.3		
451 000‐550 000	4	4.2		
551 000‐650 000	2	2.1		
651 000‐750 000	3	3.1		
>750 000	6	6.3		
Unknown	33	34.4		
Number of referrals received per month (92)				
<10	7	7.6		
11‐15	6	6.5		
16‐20	5	5.4		
21‐25	7	7.6		
26‐30	6	6.5		
>30	61	66.3		
Ages of children seen by the therapy team (97)				
0‐5 y	93	95.9		
6‐11 y	91	93.8		
12‐16 y	91	93.8		
17‐25 y	66	68.0		

(b)				
Variable (*n* responses)	Questionnaire respondents (*n* = 110)			
	Median	IQR	Min	Max
Number of therapists in the therapy team (99)	11	16	1	92
Full‐Time Equivalent (FTE) staff in the therapy team (93)	8.6	11.6	0.70	72.0
Number of children registered with the therapy team (73)	600	949	30	8000
Number of weeks wait for an initial appointment single value reported (50)	9.5	10.0	1.0	30.0
Percentage of ‘did not attend’ appointments single value reported (55)	10.0	15.5	0 0.0	55.0

The samples of pre‐appointment written materials were analysed in two stages, using an adapted framework analysis.^12^ The initial framework was developed inductively. All sample documents were read, and all content identified and labelled. Labels representing similar content were given a descriptive heading in the framework and corresponding content was categorised under the headings. Headings changed and developed to represent broader conceptual categories as documents were re‐read and analysis developed (eg see Table 2). Comments and note taking were documented in the framework to track changing categories and headings. All documents were re‐read until the conceptual headings held and all content was represented. TR reviewed the framework throughout analysis and conceptual categories and headings were discussed and agreed between SA and TR. Categories containing large volumes of, and conceptually rich, content were prioritised for further in‐depth analysis. Research memos^13^ were used to prioritise categories and explore conceptual ideas, with ‘expectations’ found to be a central concept in the materials (see Table 3). Enrolment,^14^, ^15^ defined in the sociology of translation^14^, ^15^, ^16^ as the negotiation of interrelated roles and actions to expected identities and roles, became conceptually important to understand expectations in the materials. The sociology of translation^14^, ^15^, ^16^ was subsequently used to inform the analytical framework and further in‐depth analysis and interpretation.

**TABLE 2:** Example of qualitative analysis framework development

	Early phase of analysis	Later phase of analysis	Final analysis
Category heading and description	Health Content related to: the child's medical condition	Health Content related to: the child's medical condition; health (WHO ICF domains); health professionals	Health Content related to: the child's medical condition; health (WHO ICF domains); health professionals, parent health
Example of content extracted	Does your child have any other medical conditions? For example asthma, diabetes Does your child have a diagnosis? Has your child had acute vomiting/diarrhoea? Hip scan referral?	What activities does my child have difficulty doing? Please tell us about your [child's] sleeping patterns. Please tell us about your eating habits, your feelings and other experiences you may be having Are any other professionals involved with your child's care?	As a carer do you have any health, social needs that you would like us to be aware of? Any complications during pregnancy? Birth delivery?

**TABLE 3:** Expectations as a central concept in samples of pre‐appointment written materials

Questions for further development	Related data categories
Whose expectations are they? Who is the focal ‘actor’^37^ amongst the numerous expectations?	Parent engagement Appointment information
How are expectations shared? What interactions are used for expectations to be expressed and understood?	Formatting Lexical choice/language/tone Carer information
What do the expectations relate to? Expectations about what? What events and responses are used in the content to shape expectations?	Appointment information Interventions Parent engagement

Data were subsequently framed from the theoretical perspective of the sociology of translation. Changes included re‐defining categories to represent specific entities^14^, ^16^ found in the materials. For example, ‘parent engagement’ as a category was re‐defined as ‘the parent’ and data from the materials that represented characteristics, intentions, roles, actions and behaviour of the parent was grouped in this category. Entities found in the materials were also inanimate, for example ‘the appointment’ and given their own category heading containing data on characteristics, intentions and roles of the appointment. ‘Enrolment’ was added to the framework to represent the process by which the materials shaped expectations. Content in the ‘enrolment’ category was found to include specific techniques to modify behaviour towards that expected. The techniques were analysed further using the behaviour change taxonomy^17^ and subsequent mechanisms of action.^18^, ^19^ Memoing^13^ was used in the final stage of analysis to identify key entities, actions and interactions in the data and relationships to the expectations central to pre‐appointment materials.

## 3 RESULTS

**FIGURE 1:**
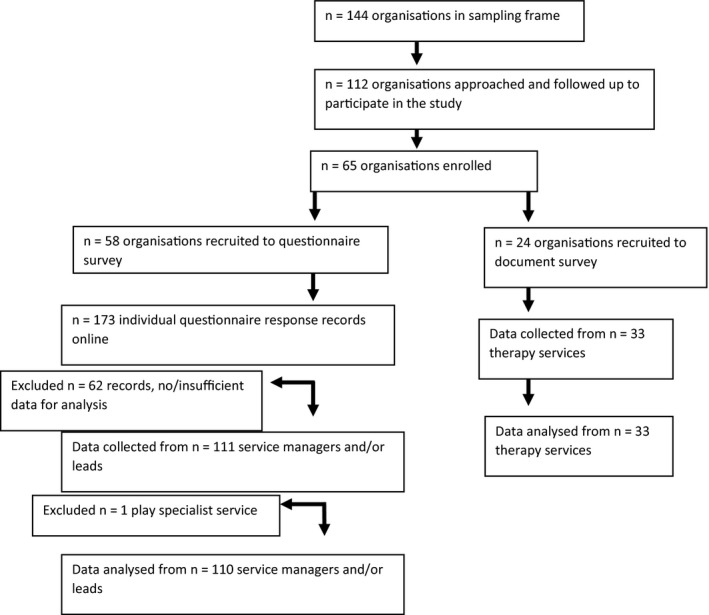
Flowchart of enrolment of organisations and participants

Of the *n* = 112 NHS provider organisations approached, data were returned for *n* = 58 organisations (response rate 51.7%) by *n* = 110 service managers and/or clinical leads. A flowchart of participating organisations and therapy services is presented in Figure 1. Sample characteristics are provided in Table 1a, b, and in an online database of children's therapy services.^7^ Participating organisations included NHS Trusts and NHS commissioned community interest companies and social enterprise organisations (England), Health Boards (Scotland and Wales), and Health and Social Care Trusts (Northern Ireland). No evidence of non‐response bias in respect of UK nation was found. No further analysis of non‐response bias was possible due to the total number of therapy services within participating organisations being unknown. All recorded responses represented data from different services, no duplication of service was found.

From all 110 survey respondents, 104 (94.5%) respondents reported their therapy service delivered pre‐appointment written materials to children and families, suggesting it to be a very common intervention in children's therapy services. The most common pre‐appointment materials were letters (*n* = 101, 32.5%), leaflets (*n* = 71, 22.8%) and questionnaires (*n* = 48, 15.4%). This was also reflected in the sample materials submitted for qualitative analysis: letters (*n* = 25), leaflets (*n* = 21), questionnaires (*n* = 12), a therapy charter (standards of practice) for parents and therapists (*n* = 1), checklists and screening forms (eg contra‐indications for hydrotherapy)(*n* = 1), consent forms(*n* = 1), referral guidance and activity pack(*n* = 1), hospital passport (a document about the child and their needs^20^) (*n* = 1) and a parent's guide (to Ponseti treatment for babies with clubfoot) (*n* = 1).

The overall, integrated results from the analysis of both the questionnaire and pre‐appointment written materials are presented as a tabulation in Appendix S1, according to the Template for Intervention Description and Replication.^8^ Appendix S1 presents the quantitative questionnaire results for each TIDieR^8^ domain alongside summary statements of qualitative findings that contribute to the intervention description. The pre‐appointment materials most commonly targeted parents (*n* = 95, 58.1%). The materials’ most common purposes were to (a) help therapy teams to be efficient and make good use of their resources (*n* = 67, 20.9%), (b) improve child and/or parent satisfaction (*n* = 65, 20.3%) and (c) make services more accessible (*n* = 60, 18.8%). Decision‐making about what materials to send and when were most commonly based on referral information or organisational pathways. Delivering the intervention by alternative modes of delivery including telephone call (*n* = 67, 43.2%) and text message (*n* = 40, 25.8%) was also common.

The remainder of this section focuses on the qualitative data analysis and the enrolment that begins with pre‐appointment written materials. Quantitative data are further integrated into results where it adds context to the findings.

### 3.1 Players and layers

Pre‐appointment written materials were found to present a complex network of people and interactions related to children's therapy. Most of the content in the materials related directly to the child, the parent and the therapist. However, they also introduced numerous other individuals and groups of people including teachers, special educational needs coordinators, headteachers, Trust chair and chief executives, patient advice and liaison advocates and, occasionally, public bodies, for example the National Institute for Health and Care Excellence. The therapy service was typically placed at the centre of the network, as a connector that introduces and links individuals and groups to the therapy context and establishes associations with children's therapy.

The written materials contained varied and multiple layers of content, all relating to the operational context of the therapy service. The core layer of content was pragmatic with a focus on the logistics of the appointment, that is the date, time and location, and the therapy service, that is profession(s) and contact details. This core content was often built upon with increasing layers of detail about the specific aspects of the appointment (eg what to bring, who will be there), the therapy service (eg opening times and team members), the organisation (eg smoking policy and parking) and the help available (eg treatments and groups).

Pre‐appointment written materials were found to enrol^14^, ^15^ the child, the parent, and the therapist to identities, roles and actions that align with the therapy service's notion of therapy and subsequently constructed service operations and procedures. Enrolment^15^ is by means of techniques (eg instruction on how to perform the behaviour^17^) that target parents’ capabilities, social opportunities and motivation^21^ (see Table 4) to align parental behaviour with the behavioural expectations of the therapy service. Lexical choices are used alongside the techniques to add salience to actions, all of which can be found in the varied layers of content. The materials, by attempting to modify behaviours, can be considered an intervention and from survey findings a common intervention. The child, the parent and the therapist are enrolled to follow the service's operational systems and procedures related to accessing the service, decision‐making about care and help‐giving.

**TABLE 4:** Behaviour change ingredients and mechanisms in pre‐appointment written materials

Behavioural factor^21^	TDF domains^22^	Intervention type	Relevance of domain	Behaviour change techniques	Statement(s) from current pre‐appointment materials
Physical capability	Physical skills	Training	Accessing the service Help‐giving practices	4.1 Instruction on how to perform behaviour 6.1 Demonstration of the behaviour 8.1 Behavioural practice/rehearsal	‘The exercises and activities will be shown to you so that you may continue, if you wish…’ ‘Sometimes it is difficult to remember information you might be asked for or questions that you might want to ask at your first appointment. Before your first appointment it may be useful to make a few notes.’
Psychological capability	Knowledge	Training Education Persuasion	Accessing the service Decision‐making about care Help‐giving practices	4.1 Instruction on how to perform behaviour 5.1 information about health consequences 13.2 framing/re‐framing	‘…contact the department more than 48 h in advance. If you have any queries or want to re‐arrange the appointment please contact the department on the above number, any weekday between 8.30 am‐12.00, 1.00‐3.00 pm’ ‘Regular attendance is essential to the progress and effectiveness of your child's treatment’ ‘At the end of the assessment, the therapist will discuss your child's difficulty with you and indicate the level of therapy needed. However, some children will not require any further appointments.’ ‘Supporting children in this way has been shown to make a significant difference to their communication skills’ ‘Follow the advice and practise any home exercises your therapist suggests. These are designed to help your child's condition’ ‘During the course of therapy, you may be given activities to practice with your child. These will form a vital^a^ part of your child's therapy.’
	Cognitive and interpersonal skills	n/a	n/a	n/a	None
	Memory attention and decision processes	n/a	n/a	n/a	None
	Behavioural regulation	n/a	n/a	n/a	None
Physical opportunity	Environmental context and resources	n/a	n/a	n/a	None
Social opportunity	Social influences	Persuasion Incentivisation	Accessing the service	6.2 Social comparison 6.3 Information about others approval 10.4 Social reward	‘Due to the constant demand for [therapy] appointments, failure to attend two or more consecutive appointments without notification may result in the session being offered to another child.’ **‘This Service is in high demand** therefore it is vital that you make every effort to attend your scheduled appointments’ "I found the appointment extremely informative and helpful." *‐parent* "Really useful information to aid us as parents to help our son." *‐ parent* ‘It is also your opportunity to have a face to face contact with a specialist [therapist]’
Reflective motivation	Professional/social role and identity	Persuasion	Help‐giving	13.2 framing/reframing	‘It is your responsibility to follow and carry out any advice and / or exercises recommended to you.’
	Beliefs about capabilities	n/a	n/a	n/a	None
	Optimism	n/a	n/a	n/a	None
	Beliefs about consequences	Persuasion Education	Accessing the service	5.2 salience of consequences 5.5 anticipated regret 9.1 credible source 16.3 Vicarious consequences	‘Last year over 1000 appointments were cancelled at a cost of £21,000.’ ‘The course is popular and can book up quickly – if there are no spaces left when you reply you will have to wait for the next block of sessions’ “My child achieved doing his shoelaces on and off his feet and will practice the double knot. He is using cutlery more confidently” *‐ parent* ‘Interventions which include CBT are recommended for generalised anxiety disorders in children and young people (National Institute for Heath and Care Excellence, NICE Pathways, 2017).’ ‘Group CBT Is a recommended treatment for social anxiety in particular. These recommendations are based on research.’ ‘Contact the therapy service if you cannot attend the appointment. This will allow us to give the appointment to another child on the waiting list.’
	Intentions	n/a	n/a	n/a	None
	Goals	Enablement	Accessing the service Decision‐making about care	1.3 goal setting (outcome)	‘Please complete the section of the leaflet focusing on what your child finds difficult during their daily activities and the areas you would like to focus on.’
Automatic motivation	Reinforcement	Incentivisation Coercion	Accessing the service Help‐giving	10.6 non‐specific incentive 10.8 Incentive (outcome) 13.2 Framing/reframing 14.2 Punishment	‘We encourage you to attend this prior to your appointment where possible, to enable you to make the most out of the [therapy] involvement with your child.’ ‘Once you have attended the workshop you will have the opportunity to work with one of our therapists in a series of individual appointments’ ‘This session provides essential^a^ information which will be important for future therapy provided, therefore it is compulsory^a^ that you attend.’ ‘[Group] is a great way to ask questions and find out about how to best support your child's development, in a relaxed and fun atmosphere’ ‘Failure to do so will count as DNA (did not attend) and may result in discharge from the service’ ‘If you are unable to attend on more than one occasion and do not inform us before the appointment you will be discharged.’ ‘If you should fail to be available for this appointment, without notifying us, we will assume that you no longer require an appointment and your child will be discharged’
	Emotion	n/a	n/a	n/a	None

Bold text denotes increased dose of the technique through formatting.

^a^ Denotes lexical choices to add salience to the technique.

### 3.2 Accessing the service

In their most basic form, the materials provide instructions on how to access the service. Instructions include how to make or change an appointment, when and where to turn up for an appointment, or how to address specific needs identified in the referral before an appointment will be offered. Service users are instructed to contact the service if the specific conditions set out in the materials, for example attending the appointment at the time specified, cannot be met. Such instructions are primarily targeted at the parent. Only one NHS child and adolescent mental health service instructed adolescent patients directly on how to access the service. All the materials were found to use behavioural instructions to educate^22^ primarily the parent about the social transactions needed for successful access to the service.

In rare cases, written materials contained only such core instructions as outlined above. Most commonly, even in their most basic forms, written materials stated the consequences of following, or not following, the instructions. Vicarious consequences,^17^ in the form of an appeal to altruism, were used to encourage the parent to follow the instructions. For example, one community physiotherapy letter stated:

If you are unable to attend a prearranged appointment you should ring the children’s therapy department … This will allow your appointment slot to be filled by another child.

By doing as instructed, the parent could, potentially, help another child and family access the service. Information about health consequences^17^ was also used in the materials to encourage the parent to do as instructed, for example:

Early treatment of a baby hip problem is much simpler and more successful than if a problem is identified later. Please ensure that you do attend the ultrasound appointment. (outpatient physiotherapy letter)

The positive effect of attending the appointment on treatment success and/or the child's health condition was common informational content to educate the parent and encourage them to follow the instructions in the materials. Positive consequences to performing expected behaviours were also framed as social opportunities to persuade and incentivise parents to follow the instructions, for example incentives^17^ in the form of access to future on‐going support being contingent on attending the appointment.

The withdrawal of access to the therapy service, in the form of the child's discharge, was frequently framed as an explicit consequence of the parent not following the instructions. Scheduling a consequence^17^ for not doing as instructed (ie not attending the appointment or contacting the service), whilst theoretically a punishment for performing unwanted behaviours^17^ was sometimes stated to be based on the assumption that the appointment, or treatment, was no longer needed or there were no longer ‘any concerns’, and access to therapy was therefore withdrawn. In rare cases, the threat of ‘further action’ being taken by the child's referring professional as a consequence of not attending the appointment was used to inform of potential future punishment^17^ for not accessing the service as instructed. Scheduled and/or future punishments were commonly used to reinforce^22^ the expected behaviours to parents.

The pre‐appointment written materials primarily enrolled parents to access the service as expected by increasing parental knowledge.^22^ Some materials used additional techniques to educate, persuade and coerce^22^ the parent to do as expected (see Table 4). Accessing the service as instructed and expected provided a gateway for decisions to be made about the child's future care.

### 3.3 Decision‐making about care

In the materials, the appointment is typically rendered as a highly structured and scheduled event at which the therapist will decide about the child's future care. The structure and schedule for the appointment support the therapist's decision‐making by providing opportunities for the therapist to observe and test the child, ask questions and elicit information that will allow a decision to be made. Pre‐appointment written materials provide the parent with information about the structure and schedule of the appointment:

First you will meet the therapist for a discussion …Your child will have the opportunity to explore the daily activities … At the end of the session you will receive practical strategies…. (community occupational therapy service)

The materials also provide instructions on how parents and children should prepare for the appointment, for example what to bring (eg a drink, consent form, child's red health book), what to wear (specifically by the child eg vest and shorts), and topics for discussion (eg activities of daily living, the child's communication). Occasionally, the materials may also inform and instruct the parent about the decision‐making process at the appointment:

Once the initial consultation is complete you will be asked to wait in the waiting area for a short time while the team meet together in order to consider possible management options for your situation. You will then be invited back into the consulting room where the treatment plan will then be explained and discussed in detail with you (outpatient multidisciplinary therapy service)

The use of pre‐appointment questionnaires sometimes provided an opportunity for the parent to contribute information and their views for use in the decision‐making process. Pre‐appointment materials instructed the parent to fill in forms, tick or circle difficulties on a questionnaire, discuss difficulties with the child or teacher and write them down, and chronicle the child's skill development to contribute information and views. The parent's information and views were always constricted to the skills, activities or actions of interest to the therapy service, for example gross motor skills, dressing, writing, bike riding, swallowing or speech, and to the child's difficulties and/or differences. Difficulties and/or differences were key to the therapist's decision‐making and most often identified by comparing the child to peers or to age‐ or performance‐based norms. Differences were sometimes, but rarely, considered in the form of goal setting^22^ that represented the differences the parent wished to see in the future. Some materials instructed the child to identify their own difficulties and/or differences by completing a questionnaire or thinking about goals but such interactive elements for children were not commonly found in the materials nor reported by survey respondents. Only forty‐seven (43.5%) managers and clinical leads completing the survey reported interactive elements for children, most commonly that the child could read the materials (*n* = 24, 22.2%) or answer questions (*n* = 10, 9.3%).

Pre‐appointment written materials primarily target parental knowledge about what will happen at the appointment and who is expected to do what so that the therapist can decide about the child's future care. Some materials also outlined decision‐making in terms of the timeframe and format (eg in writing, by discussion) in which the parent and the child would know the therapists’ decision. Decision‐making was portrayed as a process whereby the therapist matched the child's difficulties and/or differences to the help‐giving practices available from the service to propose a management plan. Some materials did provide the opportunity for the parent and the child to identify goals for therapy, enabling, to some degree, self‐management.^23^ None of the materials suggested opportunities for the parent and/or the child to share decision‐making with the therapist nor choose the help‐giving practices that might be best suited to them, from the range of help‐giving available.

### 3.4 Help‐giving

The final layer of content in some of the materials related to help‐giving practices of the therapy service. The nature and form of help‐giving was described as: advice (eg on ways to help the child, general fine motor skills); teaching (eg about the Picture Exchange Communication System (PECS)); strategies, activities, techniques and exercises (eg hydrotherapy, exercises and handwriting); referrals to other services; review (eg postural management clinics); groups (eg information sessions, developmental play and positions); courses (eg sensory courses for parents); workshops (eg anxiety management, protective behaviours, activities and skills); and therapist recommendations (eg equipment). Techniques used by the therapist to deliver help to the parent and the child were primarily described as demonstrating and modelling^22^ activities and exercises to the parent, the child and/or other key people in the children's therapy network (eg the teacher) to target the capacity and skills of people in the therapy network. The help‐giving educated and trained^22^ the adults around the child, so that ‘appropriate’ help was given to the child. To motivate the parent and the child to continue to do the activities and exercises at home, additional techniques were found in the materials. Incentives^17^ in the form of future positive improvements in the child's health were used to incentivise patients to follow the advice given and practice exercises, techniques and strategies at home. Indications of how the help would make a difference to children's skills were used to provide information about health consequences,^17^ and the parent's and child's actions in the context of the help‐giving were framed/re‐framed^17^ as a vital part of the child's therapy. Specific lexical choices, for example appointments being described as ‘essential’ and ‘compulsory’, were also found to add salience to specific aspects of help‐giving.

The materials that contained content about help‐giving also informed parents of an end to the help, and often specified the number of sessions or other key factors that would signify the end of help‐giving.

### 3.5 Configuring the child, parent and therapist to the therapy service

Pre‐appointment written materials seek to configure the child, parent and therapist to the therapy service. That is to define the identity, roles and future actions of the child, parent and therapist^16^ as part of a delineation of the therapy scenario.^16^ Rich descriptions of the therapist, child and parent roles within the therapy scenario were found. The therapist was frequently portrayed as specialist and expert, a position from which the therapist could make decisions about the child's care and direct and coordinate the roles and actions of others in the therapy network, for example observing and testing the child in the skills of interest to the therapy service, advising, recommending and demonstrating ‘appropriate’ help‐giving practices. The parent supported the therapist in their role by raising concerns about the child, asking and answering questions, reporting the child's difficulties and differences, identifying priorities and goals, and providing consent for the therapist to fulfil their role. The child's role comprised of completing the tests or activities set by the therapist, trying new things set by the therapist (eg equipment or strategies), exploring new opportunities, and in a few cases asking questions and identifying aims and goals for therapy, all of which was set in the context of having fun. The parent and the child role also included thinking about the strategies, activities and exercises given to them by the therapist and figuring out how to carry them out at home, making the therapy relevant to their home and family context, trying new things, and completing the activities, programmes and exercises given.

By highly structuring everyday operations, for example the appointment, the therapy service routinely directs the interactions between key players in the therapy network and the balance of agency and control for the management of the child's health. Current therapy structures and the pre‐appointment written materials used to convey to and enrol the parent and the child to the expected structures perpetuate the therapy services’ notion of therapy which does not appear to support or address important health concepts such as family empowerment and self‐management.

## 4 DISCUSSION

The findings from our study support a key criticism of current pre‐appointment materials used in healthcare more broadly, that the materials are primarily concerned with what services want, expect and need from patients, rather than patients’ health and care needs.^24^ Pre‐appointment written materials such as letters, leaflets and questionnaires are a seemingly ‘simple’ and routine part of care delivery in a complex system of health care. Viewed as an intervention, the materials consist of a limited number of techniques to modify patients’ behaviours related to the service's operational systems and procedures and can be considered a series of essential transactions which grant patients access to therapy. Changing written materials to be an intervention based on children's and families’ health care needs potentially provides an opportunity to target health behaviours and broader health outcomes such as family empowerment and self‐management. Incorporating intervention components and behaviour change techniques that support more personalised care may be one way to change pre‐appointment written materials to a more planned, targeted, health‐based intervention.

Personalisation is, in essence, a series of interactions between the child, parent and therapist through which goals and actions to guide care are collaboratively agreed.^23^ NHS England ^25^ propose six evidence‐based components to personalised care: shared decision‐making, personalised support planning, enabling choice (including legal rights to choice), social prescribing and community‐based support, supported self‐management and personal budgets.

From the six standard components of personalised care,^25^ two of the components—enabling choice and personalised support planning—were found in the samples of current written materials but both components were limited by the therapy service. Parent choice was identified but within the parameters of the skills, activities and behaviours of interest to the therapy service. Parents were expected to identify difficulties and/or differences their child had, which some materials suggested would form priorities and goals for intervention, giving parents some potential choice over the focus of therapy. However, all materials placed boundaries on parents’ choice by specifying or giving examples of the type of difficulties and/or differences that would be in scope for the service. Enabling parents and children to choose the focus of the children's healthcare without being restricted to bounded skill sets (eg fine/gross motor skills), isolated activities (eg handwriting or bike riding) or specific behaviours (eg managing anxiety) could support the identification of more meaningful goals and target broader health outcomes such as parental health and empowerment as well as children's health. Simple modifications to the materials, for example incorporating questions such as ‘What's the most important thing to you right now?’ from coaching interventions^26^ and explicit goal setting and action planning techniques^22^ would enable more choice and personalised care as well as facilitate timely co‐ordination of multidisciplinary care.^27^, ^28^

Some personalised support planning was also identified in the materials. Help given in the form of advice, strategies, teaching and modelling could be personalised by children and families tailoring the help to be of relevance to their own context and situation. Children and parents could also consider and identify what they personally wanted to gain from help given in the form of workshops, courses and groups, thereby personalising, to some degree, the outcomes of pre‐determined help‐giving forms. The help‐giving itself, however, did not appear to be personalised around the child, parent and family. Some materials suggested that the help given by the therapy service would be ‘appropriate’ to the child's difficulties and differences, and therapists could ensure that other adults around the child also gave ‘appropriate’ help. Within a framework of personalised care, patients are best placed to identify appropriate support based on their unique strengths and resources, shifting care planning from the ‘expert’ healthcare professional to patients who are experts by experience in their own context. Incorporating behaviour change techniques, for example social support and valued self‐identity^22^ into written materials may support families to identify strengths and resources that could facilitate change and develop written materials as a health‐based intervention.

No evidence of shared decision‐making was found in current materials. In fact, decision‐making about the child's future care was firmly situated as a core role of the therapist. The nature, level of, and access to help was positioned as decided by the therapist or group of health professionals at the appointment and the parent was told of the decisions related to future help or, at best, included in a discussion about the therapists’ decision. Little evidence of the child's right to choose was found. Indeed, it was not standard practice to address materials to the child or provide interactive elements to meaningfully involve children in decision‐making about their care. Whilst shared decision‐making has a well‐established evidence base within other sectors of children's health care, for example immunisations and acute respiratory tract infections,^29^ its implementation within children's therapy is scarce. Exploring the use of decision support tools ^29^ and questionnaires focused on patients’ values and preferences^30^, ^31^, ^32^, ^33^ related to health and treatment options, rather than children's difficulties and differences, may support shared decision‐making from the start of the therapist‐parent‐child relationship during pre‐appointment written interactions.

Finally, our study found that, commonly, patients’ who do not follow the series of transactions specified in current pre‐appointment written materials risk discharge from the therapy service based on common assumptions that the parent no longer has concerns, or the therapy is no longer needed. Smith^34^ has argued that, in public services, an interpersonal view of human behaviour, rather than a transactional view, is required. Such transactions, whilst believed to be key to efficient services, do not work for people and the inevitable variation in public services.^34^ Adopting a more interpersonal view of human behaviour^34^, ^35^ may lead to a different set of assumptions whereby those who do not follow the transactions specified may be the families most in need, or in crisis, who require more relational care^35^ and suspension of the ‘rules’ for a more personalised pathway to change.^34^ Changing the assumptions upon which the content of pre‐appointment materials is based requires system‐level change, driven through organisational policy and culture. Only through more adaptable healthcare systems will high quality, effective care, optimum health outcomes and meaningful service efficiency be realised.^34^

### 4.1 Limitations

Our sampling strategy successfully captured a breadth of data from across the UK using a first‐of‐its‐kind explicit sampling frame of children's therapy services,^7^ and resulted in a reasonable survey response rate (see Figure 1). However, the possibility remains that those who responded were more engaged with pre‐appointment materials than those who did not take part. Our study found wide variation in questionnaire results for variables related to the therapy service context. It may be that the variation reflects respondent characteristics rather than true variation between therapy services. For example, data variation may reflect the managerial level of the respondent, for example manager of a single therapy team versus manager of all therapy services within the organisation, rather than the true variation of the construct. Due to the potential sensitivity of some questionnaire variables which reflect the services’ performance against national standards, for example waiting times, the threat of reporting bias was considered. However, our findings are consistent with other data in this area^36^ increasing confidence in the reliability of performance‐based data. Eighty‐four (86.6%) survey respondents participating in our study reported delivering pre‐appointment interventions using different modes of delivery, that is telephone call, text message and emails. These alternative modes of intervention delivery are not captured in this study, limiting the generalisability of the results.

## 5 CONCLUSION

Our study has identified intervention techniques and processes used in current pre‐appointment materials. The findings suggest that services could consider using a broader range of behaviour change techniques in pre‐appointment materials to more effectively support health‐related actions and enable outcomes. Core assumptions and beliefs, for example about ‘effective’ child‐parent‐therapist healthcare interactions that underpin the content of current materials need to be challenged to support change in the discourse and the materials. Research focused on system‐level factors, for example organisational policies and routine care ‘pathways’, that drive discourse and behaviour in health care is needed to progress meaningful implementation of national policy such as personalised care.

## CONFLICT OF INTERESTS

The authors have no conflict of interests to declare.

## Funding information

This work was funded by the UK Occupational Therapy Research Foundation, a division of the Royal College of Occupational Therapists, Devices 4 Dignity and the Council for Allied Health Professionals Research.

## Supporting information

Appendix S1

## Data Availability

The data that support the findings of this study are openly available in figshare: Database of children's therapy services in the UK. Newcastle University. Dataset. https://doi.org/10.25405/data.ncl.10265012.v2.
