# Supplementary material for: Describing pre‐appointment written materials as an intervention in the context of children’s NHS therapy services: A national survey
Source: Health Expect. 2020 Dec 10;24(2):386–98. doi: 10.1111/hex.13180 (PMC8077103; doi:10.1111/hex.13180)
Supplement: Supplementary file 1 — Appendix S1 [file HEX-24-386-s001.docx]

**APPENDIX S1: Pre-appointment written materials description**

For open access to the study questionnaire and statistical analysis plan:

[https://data.ncl.ac.uk/articles/Database_of_childrens_therapy_services_in_the_UK_v1/10265012](https://data.ncl.ac.uk/articles/Database_of_children_s_therapy_services_in_the_UK_v1/10265012)

***All questionnaire items were multiple response unless otherwise indicated in the table. Multiple response results are presented as ‘percent of responses’.**

| Item number | 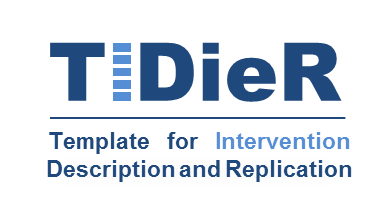Item |
| --- | --- |
| 1. | **BRIEF NAME: Provide the name or a phrase that describes the intervention.**  Pre-appointment written materials |
| 2. | **WHY: Describe any rationale, theory, or goal of the elements essential to the intervention.**  Ninety-five (86.4%) respondents provided data about the perceived advantages of pre-appointment written materials.  Survey question: What advantages do you hope that sending written materials has?  From pre-coded multiple response options, the perceived advantages were:   - help therapy teams to be efficient and make good use of their resources (n=67, 20.9%) - improve child and/or parent satisfaction (n=65, 20.3%) - make services more accessible to children and parents (n=60, 18.8%) - save clinical time (n=50, 15.6%) - help the service to comply with laws and policies (n=38, 11.9%) - improve child and parent health (n=22, 6.9%)   Eighteen (5.6%) respondents reported ‘other’ perceived advantages in open text responses. These advantages were:   - planning and individualising the child’s initial assessment - setting, meeting and managing expectations - supporting informed decision-making about the services - building positive face-to-face contacts. |
| 3. | **WHAT materials: Describe any physical or informational materials used in the intervention, including those provided to participants or used in intervention delivery or in training of intervention providers.**  One hundred and ten (100%) respondents provided data about the type of pre-appointment materials their service posts.    Survey question: Do your team post any of the following written materials to a referred child’s address before their first (assessment) appointment?  The most common materials were:   - letters (n=101, 32.5%), - leaflets (n=71, 22.8%), - questionnaires (n=48, 15.4%) - location maps (n=43 13.8%)   The full range of materials are represented in Fig A1 below.  **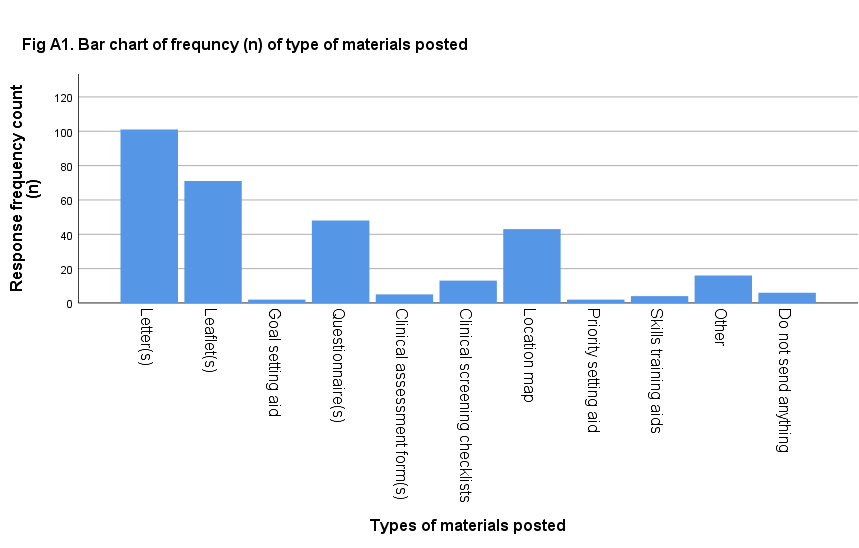**  **.**  Additional variations of pre-appointment written materials reported as ‘other’ in free text responses were:   - website links - transition documents - personal details and demographic forms - case history form and care agreement - car parking details - activity suggestions and exercises   The modal value for the total number of materials sent pre-appointment was two (n=29, 26.4%, range 8).  Ninety–one (82.7%) respondents provided data about whether and how children could interact with the pre-appointment materials.  Survey question: Can children interact with the materials in any of the following ways?  From pre-coded multiple response options, most materials were reported to contain ‘no interactive elements for children’ (n= 61, 56.5%).  When interactive elements were reported, children could:   - read the materials (n=24, 22.2%) - answer questions (n=10, 9.3%) - colour pictures (n=2, 1.9%) - draw (n=1, 0.9%)   Three (2.8%) free text responses identified ‘other’ interactive elements and described a pre-referral pack of activities for coordination or fine motor difficulties, communication symbols, and faces for the child to tick how they feel in relation to the appointment. |
| 4. | **WHAT procedures: Describe each of the procedures, activities, and/or processes used in the intervention, including any enabling or support activities.**  **4.1 Producing the pre-appointment materials**  Ninety-seven (88.2%) respondents provided data about the materials and resources their service used to produce pre-appointment written materials.  Survey question: Have you used any of the following to create and produce the materials that you post?  From pre-coded multiple response options, pre-appointment written materials were produced using:   - Trust policy (n=71, 15.6%) - staff experience (n=57, 12.5%) - parent feedback (n=53, 11.6%) - referral pathways (n=45, 9.9%) - patient information guidelines (n=28, 6.1%) - recommended templates (n=25, 5.5%) - clinical guidelines (n=23, 5.0%) - research evidence (n=23. 5.0%) - children’s feedback (n=22, 4.8%) - clinical governance team (n=18, 3.9%) - patient advocates (n=17, 3.7%) - design expertise (n=10, 2.2%)   Respondents also described using Microsoft©/computer software (n=50 11.0%) and specialist mail management software (n=3, 0.7%) to support the production.  Eleven (2.4%) respondents reported ‘other’ production materials and resources in free text responses: Trust IT department; models of practice i.e. “care aims language”; health literacy, accessible information team and easy read guidelines; Scottish Health Council advice; and feedback from schools and education.  Therapy services would most commonly use three (n=21, 19.1%, range 10) resources in combination to produce pre-appointment written materials.  The results imply a process of blending ideas and content from a range of sources and use of technical resources to support production. The range of contributors to the materials can be considered stakeholders in the intervention.  **4.2 Delivering the pre-appointment materials**  Ninety-five (86.4%) respondents provided data about the decision-making related to delivering pre-appointment written materials. Most respondents cited referral information or organisational pathways.  Survey question: Do you use any of the following to help you decide what written materials to send and when to send them to the referred child’s address?  Pre-coded multiple response options identified factors to decision-making:   - the reason for the child’s referral (n=52, 21.1%) - organisational pathways (cumulative n=106, 43%) comprising referral pathways (n=48, 19.5%), clinical care pathways (n=36, 14.6%), and assessment pathways (n=22, 8.9%) - child’s health condition (n=29, 11.8%) - child’s age (n=29, 11.8%) - waiting list management (n=12, 4.9%)   Eleven (4.5%) respondents reported none of the prespecified options were used to decide what materials to deliver.  Seven (2.8%) respondents recorded free text responses to describe their decision-making process and provided additional variations:   - information on the referral e.g. if English is a second language or visual difficulties and presenting need. - no active decision making was needed as intervention delivery was standard: “we use SI (Sensory Integration) now so all pre-set and can`t be changed by clinician” “We send a standard letter inviting them to book in.”   Most commonly, therapy services would only use one (n=23, 24.2%, range 7) of the specified factors to make decisions about what written materials to post.  **4.3 Pre-appointment materials intervention process**  Ninety-one (82.7%) respondents provided data about the responses that their service requested of parents following receipt of pre-appointment written materials.  Survey question: Do you ask parents to respond to the written materials in any of the following ways?  The most common parent responses from pre-coded multiple response options were:   - book an appointment (n=48, 30.0%) - bring the materials to the appointment (n=43, 26.9%) - complete and return a questionnaire (n=36, 22.5%)   Fourteen (8.8%) respondents selected the response option ‘no parent response requested’ suggesting pre-appointment written materials were not used to initiate a series of transactions in preparation for the initial appointment.  Nine (5.6%) respondents reported ‘other’ variations of parent response:   - call to cancel, change or confirm their appointment - complete activity sheets e.g. food diaries, fine motor activity progress sheets or assessment forms and return to the therapy team - provide further information about their child in addition to the referral information - consider questions posed in the written materials in preparation for the first appointment |
| 5. | **WHO: For each category of intervention provider (e.g. psychologist, nursing assistant), describe their expertise, background and any specific training given.**  **5.1 Providers of pre-appointment written materials**  As pre-appointment materials are sent on behalf of a service, the professional composition of the service is the description of the provider. Ninety-eight (89.1%) respondents provided data about the professional composition of their therapy service.  Survey question: who works in your therapy team(s)?  From pre-coded multiple response options, the services consisted of the following professional groups:   - physiotherapists (n=57, 35.8%), - occupational therapists (n=48, 30.2%) - speech and language therapists (n=36, 22.6%)   Eighteen (11.3%) respondents reported ‘other’ professionals in free text responses: paediatricians, specialist nurses, dieticians, health care support workers, assistants, assistant psychologist, admin staff, and psychologists, all of whom might also provide the intervention.  The modal value for the number of professional groups with the service was one (n=64, 65.3%, range 3) suggesting single disciplinary teams are most common in children’s therapy services and that pre-appointment written materials are likely to relate to that specific single discipline service.  Submitted samples of written materials were also primarily representative of single discipline services. The highest number of disciplines represented in the submitted samples was two, most commonly occupational and physiotherapy services. Additional professionals in the submitted samples, not represented in survey responses, were play specialists.  Survey question: What is your role in the team?  One hundred (90.9%) respondents provided data about their professional role(s) within their service, identifying themselves as potential intervention providers or individuals with stakes in the intervention. The frequency of responses for each pre-coded multiple response role category was:   - clinical lead (n=55, 42.3%) - therapist (n=35, 26.9%) - service manager (n=31, 23.8%)   Nine (6.9%) respondents categorised their role as ‘other’ and reported their roles in free text responses as: team leader, clinical team co-**ordinator,** professional lead, and rehabilitation co-ordinator.  The modal value for number of professional roles in the service was one (n=76, 76.0%, range 2).  **5.2 Stakeholders**  Analysis of submitted intervention samples identified further people or groups of people who may not provide the intervention but are potentially stakeholders with an interest in pre-appointment written materials as an intervention. The following people or groups were embedded in the content of the materials:   - Organisation Chair - Chief Executive - Patient Advice and Liaison Service - patient experience team - universities, students - government departments - professional regulators and professional bodies   **5.3 WHO was the recipient of the intervention**  One hundred (90.9%) respondents provided data about the person(s) to whom pre-appointment written materials are addressed.  Survey question: Who do you address written materials to?  From pre-coded multiple response options, materials are most commonly addressed to:   - the parent (n=97, 58.1%) - the child (n=25, 15.0%) - teachers (n=16, 9.6%) and other education staff e.g. special educational needs coordinator (n=12, 7.2%) - other family members (n=6 3.6%)   Eleven (6.6%) respondents provided free text responses to report delivering the intervention to ‘other’ people including: GP, social workers, referrers and therapists within the child’s locality. The modal value for total number of people to whom materials are addressed was one (n=63, 57.3% range 8). |
| 6. | **HOW: Describe the modes of delivery (e.g. face-to-face or by some other mechanism, such as internet or telephone) of the intervention and whether it was provided individually or in a group.**  Ninety-seven (88.2%) respondents provided data about the alternative modes of pre-appointment contact to deliver the intervention. Thirteen (13.4%) respondents selected the response option ‘written materials are our only contact’.  Survey question: Do your team contact children and families prior to their initial assessment appointment in any of the following ways?  From prespecified multiple response options, alternative modes of intervention delivery were:   - telephone call (n=67, 43.2%) - text message (n=40, 25.8%) - emails (n=7, 4.5%) - websites (n=6, 3.9%) - face-to-face (pre-assessment) (n=6, 3.9%)   Sixteen (10.3%) respondents reported other variations of modes of delivery: choose and book phone call with admin; telephone calls/face-to-face contacts related to the clinical pathway/assessment pathway children are on e.g. MSK pathway and school assessments; contacts dependent on child’s diagnosis; telephone calls dependent on ‘opt in’ systems and parent responses; telephone contacts if communication and accessibility needs of parents identified; face-to-face contacts if parents’ visit clinical site for other appointments.  The modal value for the total number of alternative contact methods used in addition to the written materials was one (n=41, 42.3%, range 4). |
| 7. | **WHERE: Describe the type(s) of location(s) where the intervention occurred, including any necessary infrastructure or relevant features.**  Samples of materials were submitted from across the UK and from a variety of organisations and services including Child and Adolescent Mental Health Services, hospital outpatients, child development centres, musculoskeletal services, pain services, acquired brain injury services, community services and cleft palate services. The samples and survey responses suggest the intervention is delivered widely, from locations and services with varying characteristics:  Survey question: Where in the UK is your team based?  One hundred and ten (100%) respondents provided data about the location of their service from responding to pre-coded single response options. Services in England (n= 97 88.2%), Wales (n=6 5.5%), Scotland (n=5 4.5%) and Northern Ireland (n=2 1.8%) reported delivering pre-appointment written materials to children and parents.  Survey question: What type of organisation employs the therapists in your team?  One hundred and ten (100%) respondents provided data about their employing organisation from which the intervention was developed and delivered. Organisations were NHS Trusts, Health Boards or Health and Social Care Trusts (n=105, 95.5%) or community interest companies and social enterprises (n=5 4.5%) providing services on behalf of the NHS.  Survey question: What type of NHS care do your team provide?  One hundred and five (95.5%) respondents provided data about the type of care provided by their service. From pre-coded multiple response options, services providing community care (n=89, 58.6%), hospital-based outpatient care, (n=36 23.7%), universal care (n=14 9.2%) and specialist care (n=13 8.6%) all delivered pre-appointment written materials to children and parents. Survey respondents were primarily representing services who only provided one type of care (n=72, 68.6% range 3). |
| 8. | **WHEN and HOW MUCH: Describe the number of times the intervention was delivered and over what period of time including the number of sessions, their schedule, and their duration, intensity or dose.**  **8.1 Schedule for delivery of pre-appointment written materials**  Ninety-five (86.4%) respondents provided the number of weeks wait for their service which indicates the time period within which pre-appointment materials are likely to be delivered.  Survey question: If you received a new referral today for your team(s), how many weeks would you expect the referred child to wait for the first (assessment) appointment? [open response]  From single value responses (n=50, 52.6%), the median number of weeks wait is 9.50 (IQR: 10, range 29).  Fourty (36.4%) respondents provided a lower and upper value to represent a range of values for the total number of weeks wait for their service. The median lower value for number of weeks wait is 3 (IQR 7, range 18) and the Median upper value is 12 (IQR 11, range 66).  **8.2 The number of times the intervention was delivered**  Ninety-six (87.3%) respondents provided data about the number of times their service posts pre-appointment materials to a referred child and their family before the first appointment.  Survey question: How many times will written materials be posted to the referred child’s address before their initial appointment? [single response variable]  A single delivery of the intervention is most common (n=66, 68.8% range 5). However, eighty-eight (80%) respondents provided data about factors that affect variation in how many times the intervention is delivered, suggesting that the number of times the intervention is delivered is not fixed.  Survey question: Do any of the following factors affect how many times you send written materials to children and families before their initial appointment?  From pre-coded multiple response options, factors were:   - appointment changes and re-scheduling (n=80, 47.6%) - child/parent responses (n=24, 14.3%) - waiting times (n=14, 8.3%) - reason for referral (n=13, 7.7%) - child’s health condition (n=13, 7.7%) - assessment process (n=12, 7.1%) - care pathway (n=8, 4.8%).   Four (2.4%) respondents reported ‘other’ factors in free text responses: administrator resources and capacity; lost/misplaced letters; and safeguarding procedures. |
| 9. | **TAILORING: If the intervention was planned to be personalised, titrated or adapted, then describe what, why, when, and how.**  Eighty-four (76.4%) respondents provided data about how their service personalises pre-appointment written materials.  Survey question: Do you personalise written materials in any of the following ways?  The full range of tailoring can be found in figure A2 below.  **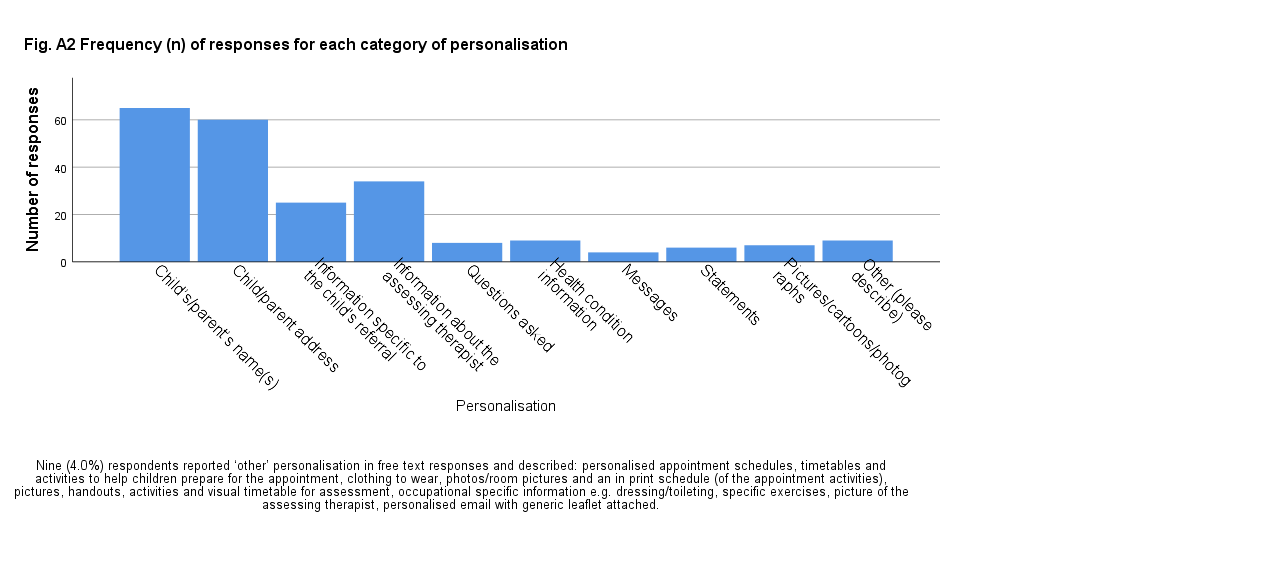**  In addition to the obvious tailoring (e.g. the services’ contact details, opening times, team members, logos) the tailored information also included:   - processes for accessing the service, - help-giving forms (e.g. therapy groups), - information about values and value statements - policies e.g. smoking policy.   Tailoring of the materials primarily reflected the service and employing organisation that delivered them. The submitted samples of pre-appointment materials provided some evidence of tailoring by service users. For example, some of the materials provided opportunity for the child and/or parent to write down their health information and history, difficulties and differences, goals or priorities for therapy, or the questions they wanted to ask at the face-to-face appointment. |
| 10. | **MODIFICATIONS: If the intervention is modified, describe the changes (what, why, when, and how).**  Survey respondents suggested reviewing the materials for potential modifications was common and provided some information about when the materials might be reviewed and modified:  Survey question: Do you review the written materials that you post? [single response variable]  One hundred and one (91.8%) participants provided data about the review of pre-appointment materials in their service. Seventy-six (75.2%) respondents reported that their therapy service reviewed pre-appointment written materials.  Survey question: What factors lead you to review the written materials you post?  Seventy-six (69.1%) respondents provided data about the factors that lead to review and modifications of pre-appointment written materials, selecting from pre-coded multiple response options. Factors were:   - changes to referral or clinical pathways (n=58, 21.6%) - child/parent feedback (n=47, 21.3%) - the amount of time the materials have been in use (n=55, 20.5%) - changes to Trust policy (n=48, 17.9%) - new/updated guidelines (n=42, 15.7%)   Eight (3.0%) respondents reported the following ‘other’ factors in free text responses: changes to the structure of the activity; how many young people are booked in and attend their first appointment; lessons learned from incidents and complaints; model of practice; new builds/location changes; staff changes; to check accuracy for information governance, and Trust mandatory review after 3 years or sooner  Submitted samples of pre-appointment materials occasionally contained the date of intervention development. One sample contained an explicit review date, suggesting a timely process of review and, potentially, modification. |
| 11. | **HOW WELL: Planned: If intervention adherence or fidelity was assessed, describe how and by whom, and if any strategies were used to maintain or improve fidelity, describe them.**  **N/A** |
| 12. | **HOW WELL: Actual: If intervention adherence or fidelity was assessed, describe the extent to which the intervention was delivered as planned**  Intervention adherence, i.e. materials sent as intended, received (e.g. post delivered, letters opened and read, parent questionnaires completed) as intended is unknown. ‘Did Not Attend’ rates at face-to-face appointments may be considered an outcome of adherence to the intervention.  Survey question: What percentage of children referred to your team(s) do not attend their first appointment? [open response]  Fifty-five (74.3%) respondents provided a single value response to report the ‘did not attend’ (DNA) rate for their service. The median value for DNA rate is 10% (IQR: 15.5, range 55). Twelve (10.9%) respondents provided a lower and upper value to represent a range of values for the DNA rate for their service. The median lower value for DNA rate is 1.5% (IQR 5, range 20) and the Median upper value is 7.5% (IQR 7.5, range 24). |
